# Institutional Notes and News

**Published:** 1911-05-06

**Authors:** 


### Institutional Notes and News.

Although there is now a deficit of £4,900, chiefly due to the building operations which have taken place during the past year, the North Ormesby Hospital's income continues to improve. Since Mr. E. C. Smith was appointed secretary two years ago, there has been a progressive increase in the workpeople's contributions, which form the basis of the hospital's income, owing to the industrial nature of the district. North Ormesby has become merged in Middlesbrough, and as the majority of the patients come from the works in Middlesbrough, it is gratifying to find that their subscriptions have again increased this year by nearly £500. The building operations referred to include the new sleeping rooms for nurses, which were necessary owing to the increased staff that the opening of the Caroline Brown wing entailed. The enlarged out-patient department received 5,386 patients, or some 1,600 more than previously, and, as a consequence, the ophthalmic department which was also built was kept busy. As a result of administrative changes, the cost per bed has been much reduced. The aim at present is to pay off the accumulated debt, so that the increased cost of maintenance which the new buildings and growing number of patients involve may be met, and it is much hoped that Middlesbrough, which, of course is the real district that the hospital serves, will again extend its number of employee subscribers. An appeal has been successfully started.

**Middlesbrough Workpeople and their Hospital.**

The question of the effects on hospitals of the medical inspection of school children was again brought forward by Dr. Pollard at the recent committee meeting of the Worcester Ophthalmic Hospital. He stated that since January 1 thirty-two children from the country and fifty-nine from the city had been treated at the hospital. These cases occupied the time and attention of the medical staff to a much greater degree than did ordinary cases, and it was not to be expected that the honorary staff could continue to devote so much of its time to these cases without assistance. The present subscription for an out-patient's letter does not cover the cost of the drugs and spectacles required for the majority of cases, and it was suggested that the subscription for an out-patient's ticket should be raised. Further consideration of the matter was deferred with a view to recommending to the Governors an alteration in the rule affecting the subscriptions for letters.

**Worcester Ophthalmic Hospital.**

The ladies of the Ladies' Guild of the London Homœopathic Hospital are busy with their preparations for a grand sale of work at the hospital in fulfilment of their promise to raise £500 towards the Extension Fund. The Duchess of Hamilton and Brandon has consented to open the sale at 2.30 p.m. on Tuesday, May 23, when it will be open till 10 o'clock. The members of the Guild hope to raise £500 for the much-needed wards for middle-class contributing patients. The Hampstead branch of the Guild is giving in the new Sir Henry Tyler wing of the hospital Milton's Masque of "Comus," with the original music by Henry Lawes. It will be performed on the afternoon and evening of Tuesday, May 23, at 5 and 8.15 p.m., under the direction of Mr. Savage Cooper. At two o'clock on the same day (May 23) the Duchess of Hamilton and Brandon will lay the foundation stone of the new Home for the nurses of the hospital, towards which another £6,000 is required to complete the cost of the building.

**Grand Sale at Homœopathic Hospital.**

An important departure in the policy of the working of the Evelina Hospital for Sick Children was made known at its annual meeting, over which Lord Duncannon presided on Friday last week. That is the appointment of an inquiry officer, in accordance with the suggestions of King Edward's Hospital Fund, and, consequent on this innovation, the establishment of a Samaritan Fund. The report states that there has been a heavy loss in subscriptions of late years, and that while the cost of out-patients has increased, in spite of smaller numbers, the figures *re* the in-patients compare favourably with those for 1909. Miss E. M. Ranking is the inquiry officer, and the constructive side of her work is much helped by the Samaritan Fund, which enables her to help the poorest patients to buy surgical appliances. The fund was started with a legacy from Mr. W. Evans and with £100 from Mr. Leopold de Rothschild. The report was adopted.

**A New Departure at the Evelina Hospital.**

The Radcliffe Infirmary and County Hospital, Oxford, adds its name to the list of these institutions which, having tentatively adopted the expedient of an almoner, have found this official invaluable to patients and hospital alike. The recent report shows that Miss Linton has found the chief scope for her work, not so much in its restrictive aspect in preventing the few well-to-do persons from applying for help, as in its constructive side, by which "cases have been followed up to their homes, that the benefit received at the hospital may be made permanent." The number of in-patients has gone up by 116, and the total expenditure has been just under £11,000. The Briscoe Bequest, for the first time having realised the full value of its investments, has enabled a new balcony to be built on to the Briscoe Ward, and several necessary improvements have been made to the laundry and ward kitchens.

**An Almoner's Work at Oxford.**

In October of last year, a sub-committee was appointed by the Board of the Northampton General Hospital to consider and report upon the question of rearranging and rebuilding the mortuary and post mortem room; also as to what use can be made of the old laundry buildings. After long and careful consideration the following scheme has been adopted, subject to confirmation by the Courts: The mortuary and post mortem room should be thoroughly restored and fitted with modern fittings and appliances, and a new room, properly furnished, should be built on to the present mortuary in which the friends of deceased patients, also coroners' juries, will be able to view the bodies. The laundry to be altered sufficiently to make of the ground floor a coal store and the upper floor a general store. The estimated cost of the whole of the work is £700. The Board thinks that the time has come when the conversion of the hand-power lifts in the new wings into electrically driven lifts might be carried out. Estimates have been obtained from three of the leading firms of lift makers in the country, and if the Court agree to this suggestion, they recommend that the tender for £545 is recommended for acceptance. July 2 has been fixed for Hospital Sunday this year, and a circular letter has been sent to the ministers and officers of all places of worship in the town, the country, and the surrounding districts, asking for their support. It is interesting to note, in view of the recent fires that have occurred at Leeds and Fazakerley, that the fire appliances at Northampton have been examined by the Borough Fire Inspector and have been pronounced by him to be efficient and in good working order. He also considers the staff are well trained in the use of the appliances.

**The New Improvements at Northampton.**

